# Hemodiafiltration Should Be the Primary In-Center Kidney Replacement Modality in ESKD: PRO

**DOI:** 10.34067/KID.0000000614

**Published:** 2025-06-10

**Authors:** Bernard J.M. Canaud

**Affiliations:** Dialysis and Intensive Care Department, Montpellier University, Montpellier, France

**Keywords:** chronic hemodialysis, CKD, chronic kidney failure, dialysis, ESKD, outcomes, renal replacement therapy

## Introduction

Emerging evidence suggests that hemodiafiltration (HDF) may provide better outcomes in KRT, prompting a reconsideration of HDF as the standard of care for in-center treatment. Although in-center hemodialysis is the most common form of KRT, sustaining millions of patients with ESKD, it has notable medical limitations, side effects, and burdens on both patients and healthcare systems.^1^ Obviously, more improvements in kidney care are still needed, despite advances in hemodialysis technique.

## Why Does Conventional Hemodialysis Remain Unsatisfactory and Why Change in KRT by Dialysis Is Needed?

Conventional in-center hemodialysis typically involves three-to-four-hour sessions, three times a week, using high-flux hemodialyzers and bicarbonate-buffered dialysis fluid. Although widely used, the mortality rate among prevalent patients (>365 days) is alarming, with an average annual death rate of 15%, ranging from 7% to 22%.^1,2^ In addition, intradialytic symptoms remain common and a source of discomfort and risk. Hospitalizations and outpatient interventions to address dialysis-associated conditions, including issues related to vascular access, are also common. The overall patient experience remains poor, with the conventional in-center hemodialysis regimen contributing to a significant burden of kidney disease.^3^

## What Goals Must a New KRT in Hemodialysis Achieve to Be Considered Superior and Initiate a Paradigm Shift?

To revolutionize KRT in hemodialysis, we can have a look to the America's Cup for inspiration. Just like a challenger in the Cup, HDF must meet or exceed six critical goals to surpass the defender, which is conventional in-center hemodialysis. The key goals for online HDF are (*1*) technical availability: ensuring safety, efficacy, and ease of use; (*2*) patient outcomes: reducing mortality, morbidity, and intradialytic symptoms; (*3*) patient experience: improving quality of life and reducing treatment burden; (*4*) cost-effectiveness: providing economic benefits without sacrificing care; (*5*) sustainability: being an eco-friendly long-term solution; and (*6*) generalizability: applicable to all patients, regardless of individual or regional differences. Evidence supporting these goals has been built over 40 years, making HDF a potential replacement for conventional.

## How Does HDF Fulfill All These Criteria through the Lens of Evidence-Based Medicine?

### Technical Availability

Online HDF requires a water treatment system capable of consistently delivering ultrapure water to the dialysis facility. Online HDF option is available in almost all hemodialysis registered machines in Europe and Japan.^4,5^ Fresenius Medical Care has received 510(k) clearance in February 2024 from the US Food and Drug Administration for its 5008X Hemodialysis System and the FX CorAL dialyzer. Several prospective studies, including long-term large national registry studies, have confirmed the reliability and safety of the online HDF therapy worldwide.

### Patient Outcomes

The recent prospective randomized controlled European CONVINCE study demonstrated that high-dose HDF, specifically, over 23 L per session three times per week, reduced the relative risk of all-cause mortality by 23% in a large population.^6^ In addition, the study showed a reduction in mortality from noncardiac causes, particularly infections, while mortality from cardiac causes tended to decrease, although not significantly. These findings align with results from the previous ESHOL study and the European HDF pooling project, which included an individual patient data meta-analysis of more than 2793 patients (HDF 50%).^7^ More recently, an updated Individual Patient Data Meta-Analysis, involving over 4153 patients (hemodialysis 2070; HDF 2083), confirmed a 16% reduction in the relative risk of all-cause mortality, 22% reduction in cardiac-related deaths, and 33% reduction in cardiac mortality.^8^ Interestingly, this *post hoc* analysis documented that mortality risk was inversely related to the convective dose delivered, confirming a dose-dependent effect.^8^ Previous Randomized Controled Trial studies have also shown a reduction in intradialytic morbidity, such as intradialytic hypotension.

### Patient Experience

The CONVINCE study developed and used innovative quality-of-life measurements, based on a novel adaptation of patient-reported outcome measurement information system with copyright^®^ tool, which relies on a web-based interactive and computerized adaptive questionnaire. This approach demonstrated that high-dose HDF mitigates the decline in various quality-of-life domains, physical, mental, and social interaction, observed in the conventional hemodialysis arm.^9^ Uniquely, the CONVINCE study longitudinally monitored these parameters over time in a self-adaptive way, offering a novel and highly personalized approach to assessing patient experience. In addition, HDF has been shown to mitigate the decline in cognitive function observed in hemodialysis patients, which may be linked to a reduction in intradialytic symptoms and the overall burden of kidney disease treatment.

### Cost-Effectiveness

Online HDF uses the same hardware as conventional hemodialysis, including HDF machines, hemodialyzers, and disposable tubing. Its clinical implementation is as straightforward as conventional hemodialysis machines, meaning that handling does not introduce additional workflow complexity.^10^ Online HDF offers features that ease caregiver tasks, such as online priming and rinsing of the extracorporeal circuit, eliminating the need for an external saline bag and allowing for an intravenous bolus during treatment. These reduce nurse workload, lower costs, minimize plasticizer residue risks, and help cut plastic waste, supporting sustainability. However, additional costs for HDF may arise from the use of sterilizing filters and microbiological monitoring of dialysis fluid, depending on local regulations and practices.

### Sustainability

Online HDF, compared with conventional hemodialysis, offers a way to reduce water and dialysis fluid consumption while increasing the clearance of uremic solutes, particularly those of middle and large molecular weight.^11^ By increasing the filtration fraction up to 35%, a proxy for convective clearance, *ß*2M clearance can be increased by up 2.5 times compared with hemodialysis, whereas total dialysis fluid production may be reduced. High-volume HDF enhances the clearance of uremic solutes by adding fully saturated ultrafiltrate to the effluent dialysate. Aligning the dialysis flow to blood flow with a ratio as low as 1.2 may also help reduce dialysate consumption. Optimized online HDF thus offers a more eco-friendly KRT capable of reducing or containing dialysate flow consumption at lower levels than hemodialysis.^11^

### Generalizability

The results from recent interventional studies comparing HDF with hemodialysis were predominantly produced in Europe, involving a relatively homogeneous population with specific anthropometric characteristics. This raises questions about the generalizability of these findings to other populations. It is now well recognized that the clinical benefits of HDF are mainly driven by the convective dose delivered, typically measured by the substitution or total ultrafiltration volume per session. To accurately compare different populations, it is advisable to scale this convective dose to patient characteristics, such as body surface area, which may serve as a metabolic proxy for resting energy expenditure. For example, in the European population studied in the CONVINCE trial, the average body surface area was 1.86 m^2^, making the 23 L of substitution equivalent to approximately 12.4 L per m^2^. Using this reference value and simple nomogram allows for the establishment of a minimum substitution volume tailored to different population profiles and substitution modality.^7^

## Time Has Come to Shift to HDF as Primary In-Center Kidney Replacement Modality

Given the limitations of conventional hemodialysis in improving patient outcomes, it is crucial to focus on HDF as the primary in-center KRT. Recent evidence clearly demonstrates the superiority of HDF when administered at optimal dosages, consistently outperforming conventional hemodialysis in key patient care metrics.

The compelling totality of evidence calls for action: physicians must adopt HDF and tailor it to their patients. The scientific community should integrate HDF into best practice guidelines. Adopting HDF as the standard of care will transform kidney replacement treatment paradigm and improve patient outcomes while reducing treatment burden.

## Supplementary Material
