# Supplementary material for: Hemodiafiltration Should Be the Primary In-Center Kidney Replacement Modality in ESKD: PRO
Source: Kidney360. 2025 Jun 10;6(10):1649–51. doi: 10.34067/KID.0000000614 (PMC12778004; doi:10.34067/KID.0000000614)
Supplement: Supplementary file 1 [file kidney360-6-1649-s001.pdf]

ASN American Society of Nephrology
JASN Journal of the American Society of Nephrology
KIDNEY360 Accessing Our World From Every Angle
CJASN Clinical Journal of the American Society of Nephrology

ASN Journal Disclosure Form

As per ASN journal policy, I have disclosed any financial relationships or commitments I have held in the past 36 months as included below. I have listed my Current Employer below to indicate there is a relationship requiring disclosure. If no relationship exists, my Current Employer is not listed.

B. Canaud reports the following:
Employer: Scientific consultant for Fresenius Medical Care, Germany up to December 2022 - Retired from December 2022; Consultancy: Senior scientist consultant for Fresenius Medical Care up to December 2022; and Other Interests or Relationships: CEO of MTX Consulting Int, Montpellier-France.

I understand that the information above will be published within the journal article, if accepted, and that failure to comply and/or to accurately and completely report the potential financial conflicts of interest could lead to the following: 1) Prior to publication, article rejection, or 2) Post-publication, sanctions ranging from, but not limited to, issuing a correction, reporting the inaccurate information to the authors' institution, banning authors from submitting work to ASN journals for varying lengths of time, and/or retraction of the published work.

Name: Bernard J. Canaud
Manuscript ID: K360-2024-000715R1
Manuscript Title: Hemodiafiltration Should Be the Primary In-Center Renal Replacement Modality in ESKD: PRO
Date of Completion: September 27, 2024
Disclosure Updated Date: May 25, 2024
